# Supplementary figures and images for: P21 activated kinase 6: a promising tool for predicting small cell lung cancer diagnosis and treatment response
Source: PeerJ. 2025 Jul 21;13:e19714. doi: 10.7717/peerj.19714 (PMC12288745; doi:10.7717/peerj.19714)

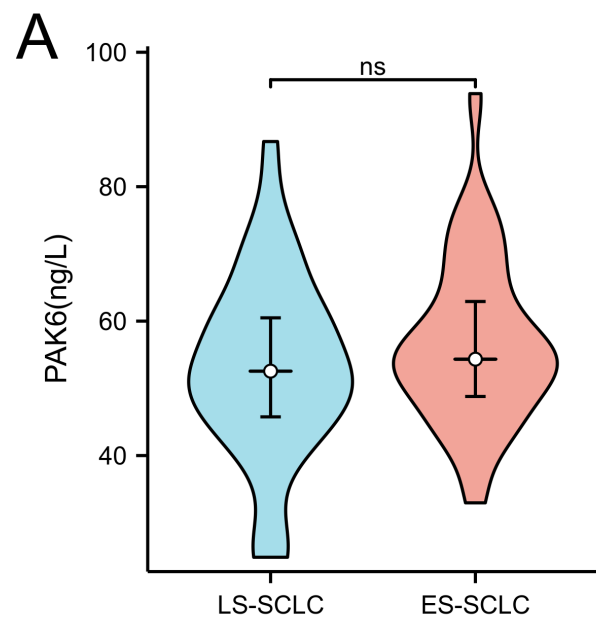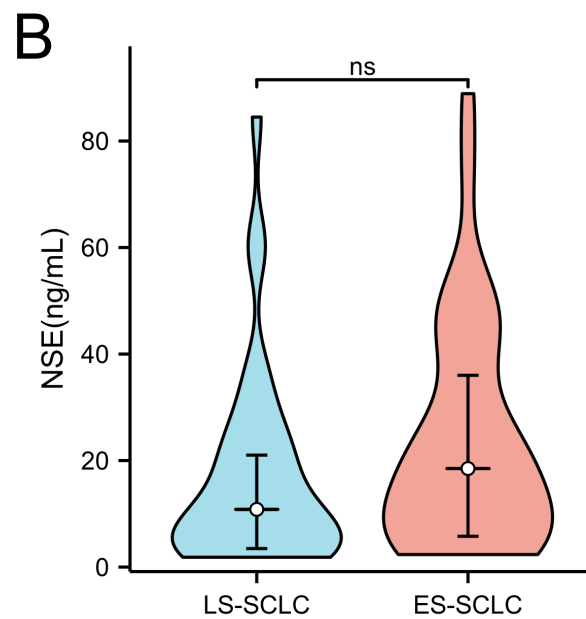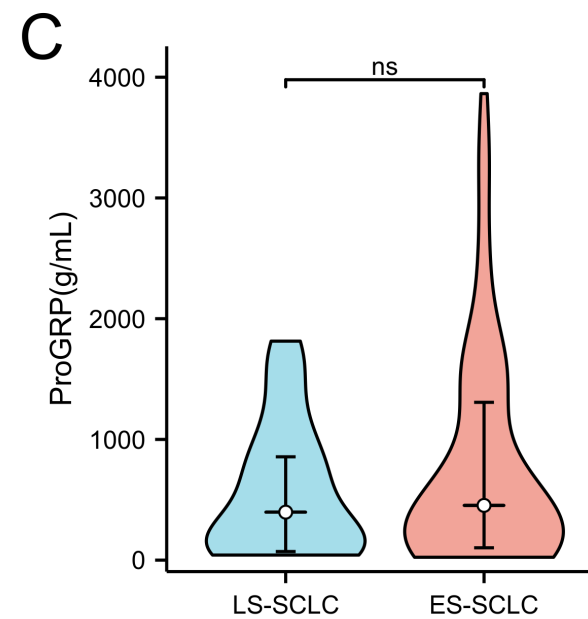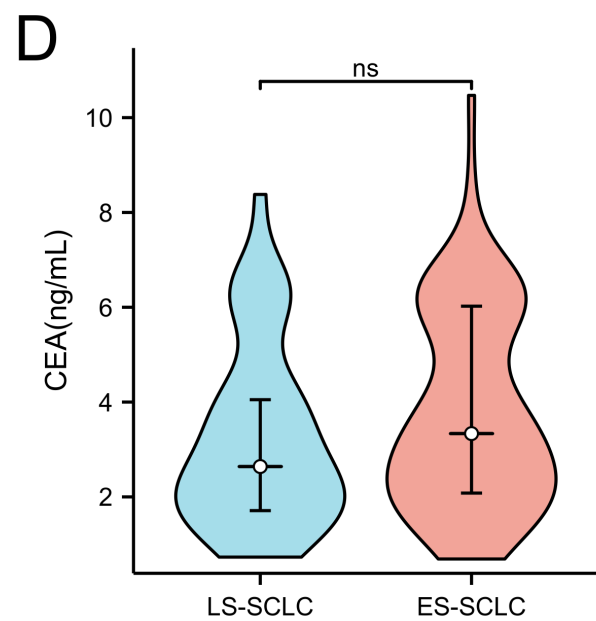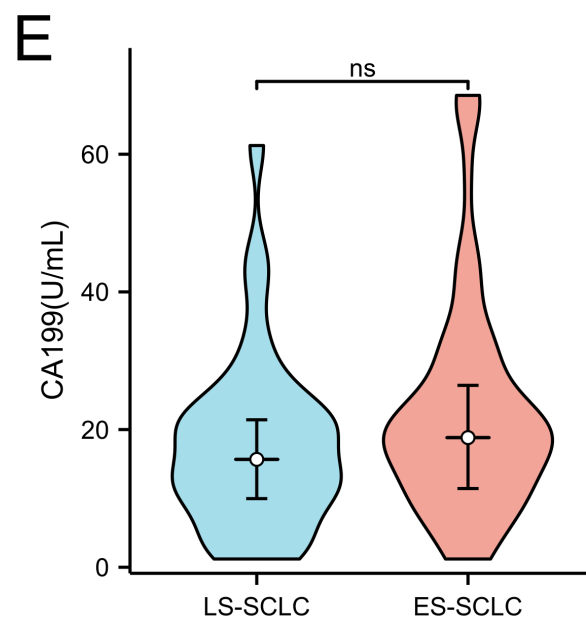

Supplement: Supplemental Information 1 — Expression levels of PAK6(A), NSE (B), ProGRP(C), CEA (D), and CA19-9 (E) in LS-SCLC and ES-SCLC groups,There was no statistical difference between all marker groups. [file peerj-13-19714-s001.pdf]

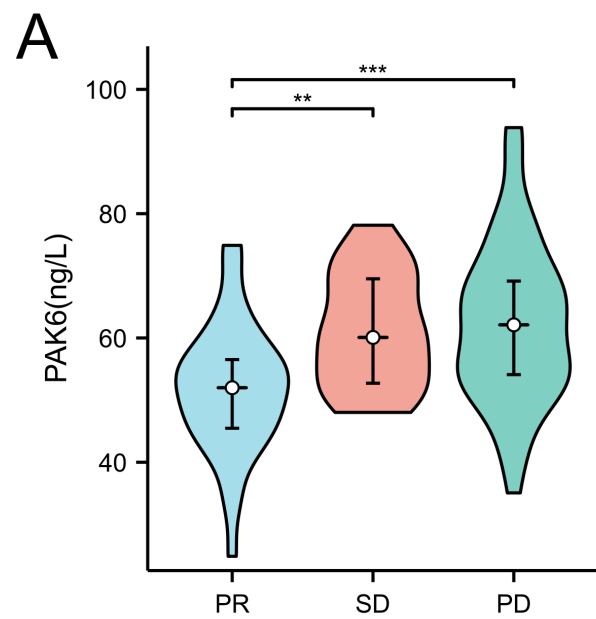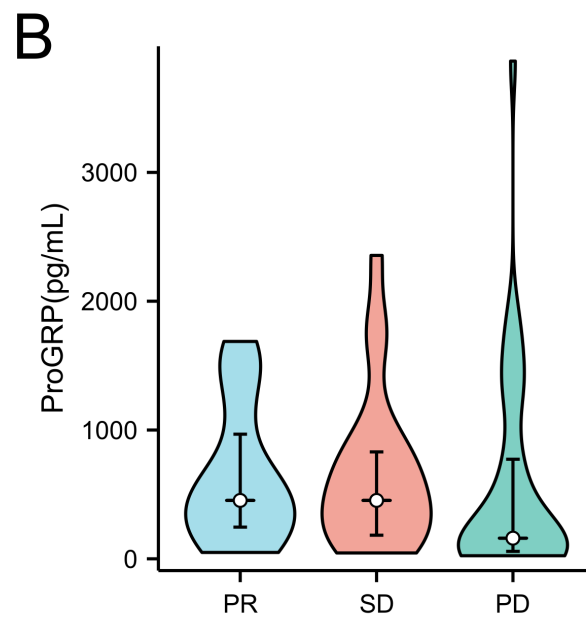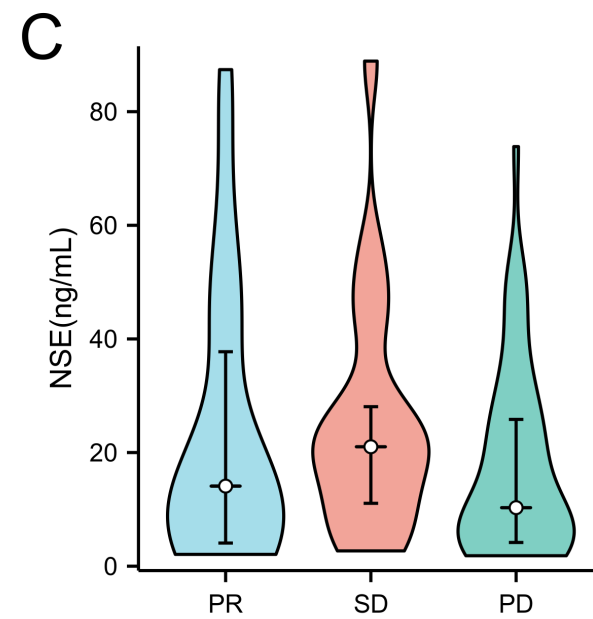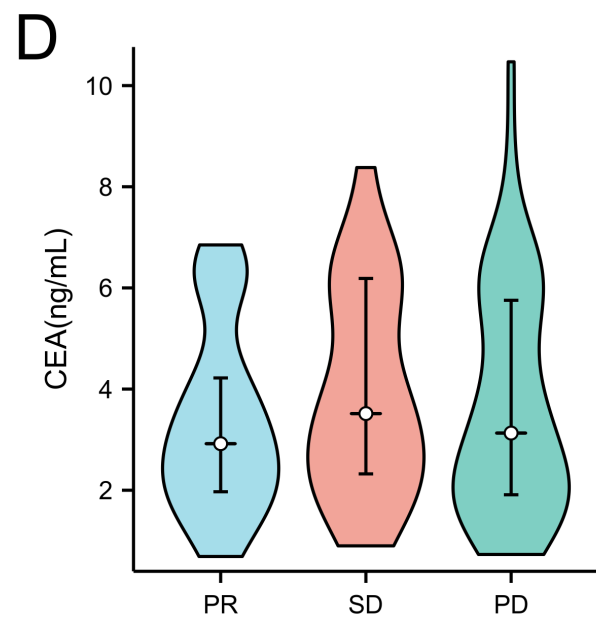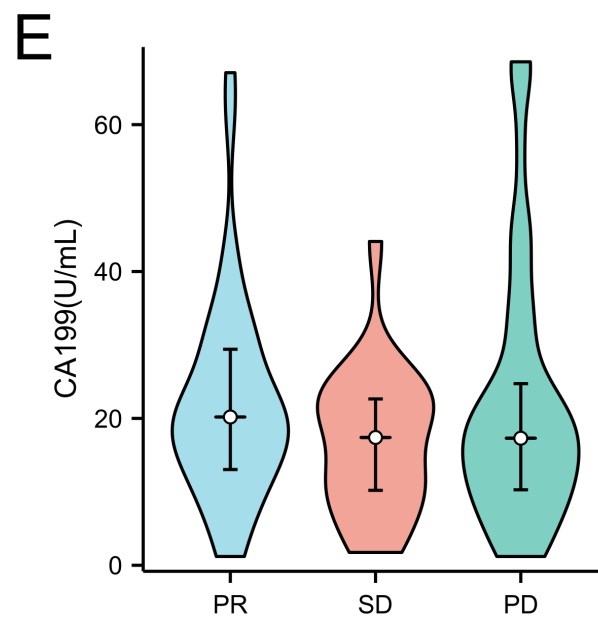

Supplement: Supplemental Information 2 — The correlation between the serum levels of PAK6(A), NSE (B), ProGRP(C), CEA (D), and CA19-9 (E) at the initial diagnosis of SCLC and the post-treatment status of SCLC, categorized as partial response/stable disease (PR/SD) or progressive disease (PD). the level of PAK6 was significantly associated with treatment response Error bars represent median and interquartile range. (∗∗P ¡ 0.01,∗∗∗P ¡ 0.001) [file peerj-13-19714-s002.pdf]
