# Supplementary material for: P21 activated kinase 6: a promising tool for predicting small cell lung cancer diagnosis and treatment response
Source: PeerJ. 2025 Jul 21;13:e19714. doi: 10.7717/peerj.19714 (PMC12288745; doi:10.7717/peerj.19714)
Supplement: Supplemental Information 3 [file peerj-13-19714-s003.docx]

**Supplementary Table SⅠ Serum marker levels in each group**

| Markers | SCLC（n=109） | NSCLC(n=94) | PN（n=85） | NC（n=94） | *P* |
| --- | --- | --- | --- | --- | --- |
| PAK6 (ng/L) | 52.48（44.50～60.61) ^a b c^ | 41.07（32.48～50.29）^a^ | 37.82（31.75～43.32） | 34.75（26.9～40.7） | <0.01 |
| NSE (ng/mL) | 8.47（3.63～  30.10）^abc^ | 3.72（3.07～4.81）^a^ | 3.13（2.42～3.81） | 3.86（3.32～4.42） | <0.01 |
| ProGRP (pg/mL) | 353.95（78.00～1224.50)^abc^ | 71.85（48.70～91.00）^a^ | 62.10（44.20～99.80）^a^ | 38.55（30.65～46.40） | <0.01 |
| CEA (ng/mL) | 2.93(2.00～4.76)^ab^ | 4.03(2.65～19.74)^ab^ | 1.29(0.82～2.39)^c^ | 1.15(0.79～1.86) | <0.01 |
| CA19-9(U/mL) | 17.89（11.39～32.44）^a c^ | 17.72（11.48～29.50）^a b^ | 11.53（6.12～19.99） | 12.32(7.29～17.79） | <0.01 |

Notes: ^a^ compared with the NC group; ^b^ Compared with the PN group; ^c^ Compared with the NSCLC group,

**Supplementary Table SⅡ Serum levels of markers before and after treatment in SCLC group**

| Markers | TREATED BEFORE(N=56) | TREATED (N=56) | *P* |
| --- | --- | --- | --- |
| PAK6 (ng/L) | 56.44(50.09～68.95)^a^ | 48.17(39.57～53.73) | ＜0.01 |
| NSE (ng/mL) | 11.73(4.14～37.37)^a^ | 4.07(2.67～7.06) | ＜0.01 |
| ProGRP (pg/mL) | 308.95(95.20～56.44)^a^ | 95.20(53.60～360.53) | ＜0.01 |
| CEA(ng/mL) | 2.91(1.92～4.66) | 2.60(2.08～3.74) |  |
| CA19-9(U/mL) | 17.78(11.72～25.91) | 16.09(11.27～30.75) |  |

Notes: a compared with treated group

**Supplementary Table SⅢ Serological levels of markers in different treatment response groups**

| Marker | PR（n=40） | SD(n=22) | PD（n=47） |
| --- | --- | --- | --- |
| PAK6 (pg/L) | 51.99(45.473-56.525)^ab^ | 60.09 (52.72-69.52) | 62.11 (54.09-69.15) |
| NSE (ng/mL) | 14.09(4.08-37.75) | 21.02 (11.07-28.07) | 10.30 (4.18-25.84) |
| ProGRP (pg/mL) | 453.40(144.72-737.85) | 453.40 183.33-830.08) | 181.40 (70.05-1042.40) |
| CEA (ng/mL) | 2.92(1.97-4.22) | 3.52 (2.32-6.19) | 3.13 (1.91-5.76) |
| CA19-9(U/mL) | 20.19（13.04-29.41） | 17.41（10.21-22.65） | 17.30（10.28-24.73） |

Notes：a represents PR compared with SD, *p* = 0.0074, b represents PR compared with SD, *p* = 0.0001.

**Supplementary Table SⅣ PFS-related data of STMs for SCLC**

| Marker | Cut-off value | Group(number) | Total censored ratio(%) | Median PFS  (days) | 95%CI of  median PFS | P |
| --- | --- | --- | --- | --- | --- | --- |
| PAK6  (pg/L) | 60.10 | Low(70) | 17.1 | 194 | 153-267 | 0.001 |
|  |  | High(39) | 5.1 | 92 | 91-123 |  |
| NSE  (ng/mL) | 3.01 | Low(13) | 7.7 | 215 | 150-？ | 0.441 |
|  |  | High (96) | 13.5 | 126 | 92-194 |  |
| ProGRP (pg/mL) | 53.1 | Low(32) | 15.6 | 99.5 | 92-246 | 0.136 |
|  |  | High(77) | 11.7 | 156 | 104-215 |  |
| CEA  (ng/mL) | 2.83 | Low(50) | 10 | 150 | 92-225 | 0.260 |
|  |  | High(59) | 15.3 | 154 | 94-214 |  |
| CA19-9  (U/mL) | 13.15 | Low(39) | 15.4 | 198 | 129-288 | 0.091 |
|  |  | High(70) | 11.4 | 107 | 92-192 |  |
